# Fatty Acid Profiles in the Gonads of Red King Crab (*Paralithodes camtschaticus*) from the Barents Sea

**DOI:** 10.3390/ani13030336

**Published:** 2023-01-17

**Authors:** Alexander G. Dvoretsky, Fatima A. Bichkaeva, Nina F. Baranova, Vladimir G. Dvoretsky

**Affiliations:** 1 Murmansk Marine Biological Institute (MMBI), Russian Academy of Sciences (RAS), 183010 Murmansk, Russia; 2 N. Laverov Federal Center for Integrated Arctic Research of the Ural Branch (FECIAR UrB), Russian Academy of Sciences (RAS), 163000 Arkhangelsk, Russia

**Keywords:** fatty acids, red king crab, ovaries, testes, Barents Sea

## Abstract

**Simple Summary:**

Invasive red king crabs support a large-scale fishery in the Barents Sea, but, currently, their by-products are not used by the food and pharmaceutical industries, partly because information regarding the biochemical content of the by-products is scarce or absent. For this reason, we assayed fatty acid profiles in the ovaries and testes of adult red king crabs. We found a predominance of polyunsaturated fatty acids and lower levels of saturated and monounsaturated fatty acids. Female gonads showed higher levels of fatty acids than male gonads due to higher levels of energy allocation to reproduction processes in females, which have larger and heavier gonads but smaller and lighter bodies than males. Ovaries are characterized by excellent quality in terms of the high content of essential fatty acids and could be recommended for consumption and as a source of valuable substances.

**Abstract:**

Red king crab (*Paralithodes camtschaticus*) is a large shelf species native to the Pacific Ocean. Due to its high commercial value, this species was introduced into the Barents Sea, where it has formed a new population that now supports a stable commercial fishery. Information on fatty acid profiles in different tissues of the Barents Sea *P. camtschaticus* is scarce. For this reason, the gonads of red king crabs collected at a coastal site in the Barents Sea during the summer were analyzed for fatty acid composition by gas-liquid chromatography. The proportions of total saturated fatty acids, monounsaturated fatty acids, and polyunsaturated fatty acids in the ovaries of *P. camtschaticus* were 25.9 ± 2.0%, 22.5 ± 2.3%, and 51.6 ± 2.5%, respectively; in the testes, these levels accounted for 35.1 ± 5.7%, 19.1 ± 2.0%, and 45.8 ± 4.5%, respectively. Fatty acid profiles were similar in larger and smaller red king crabs and crabs with old and new shells. Concentrations of fatty acids were higher in ovaries compared to testes, reflecting higher reproductive efforts in female red king crabs. High levels of docosahexaenoic and eicosapentaenoic fatty acids detected in the ovaries of red king crabs from the Barents Sea indicate that these gonads can be a good alternative source for these fatty acids in the human diet and/or for extracting important fatty acids for use in the medical and pharmaceutical industries.

## 1. Introduction

The red king crab *Paralithodes camtschaticus* (Tilesius, 1815) was transferred from Peter the Great Bay (Sea of Japan) and the Ozernovsky area, West Kamchatka (Sea of Okhotsk) to the Barents Sea by Russian scientists from 1960 to 1969 [1,2,3]. Since then, the expansion and population growth of this species have been registered both at coastal sites and offshore. From 1994 to 2001, the crab stock was exploited through research fishery conducted cooperatively by Russia and Norway [3]. Commercial harvesting of *P. camtschaticus* began in 2002 Norway and two years later in Russia [2,3]. There has been an increase in the total biomass of *P. camtschaticus* over the past decade [3,4] and, today, this new Atlantic population of red king crab supports a relatively large-scale commercial fishery in Russian waters of the Barents Sea, with total catches of 10–11.5 thousand metric tons per fishery season [5,6,7,8,9,10]. The red crab stock also supports recreational fishing in the coastal waters of the Kola Peninsula, with an annual quota of 100 t [8].

Due to its large body size and relatively high yield of tasty meat, red king crab is a high-priced species that is mainly consumed in high-end markets in the USA, Japan, and South Korea. Live red king crabs and frozen clusters that include three walking legs plus one claw are the main products on the market. However, by-products from the processing of red king crab (carapace, hepatopancreas, gonads, and hemolymph) amount to approximately 35% of the crab’s weight [11]. Thus, due to the large quantities harvested, the Barents Sea red king crab fishery generates large amounts of crab by-products. However, at present, most—if not all—by-products from this fishery are discarded and dumped at sea [12]. This situation is associated with the fact that, despite the relatively long history of red king crab studies in the Barents Sea, data on the biochemical profiles of its tissues are scarce and fragmentary. The nutritive and/or pharmaceutical value of crustacean organs is primarily determined by hormones, enzymes, and lipids, especially polyunsaturated fatty acids [11,12,13,14,15,16]. For this reason, biochemical assays of red king crab by-products are important to the chemical and medical industries.

**Figure 1:**
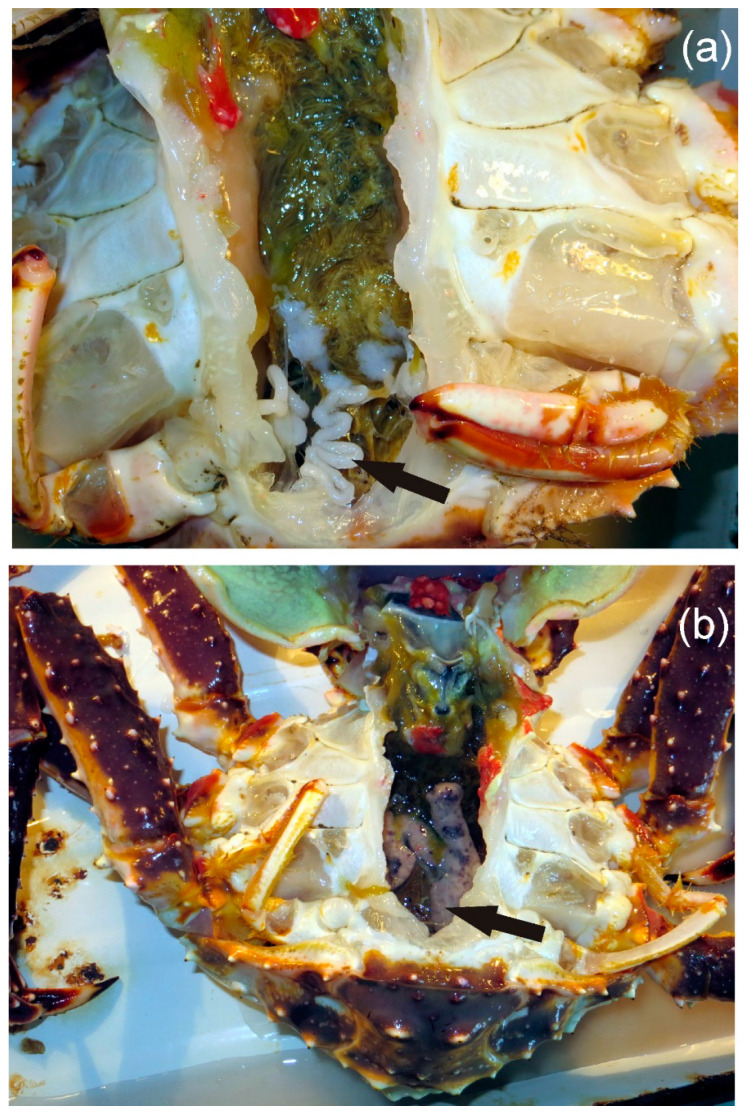
Gonads of male (**a**) and female (**b**) red king crab *Paralithodes camtschaticus*.

Fatty acid profiles of the hepatopancreas, meat, and hemolymph of *P. camtschaticus* have been presented previously [11,14,15,16], but there is no information about fatty acid concentrations in the gonads of this species in the Barents Sea. Both male and female red king crabs have paired gonads (Figure 1).

The testes are located primarily in the middle portion of the crab body, and a small portion extends into the first and second abdominal segments. The testes contain the seminiferous tubules where spermatozoa are generated. The reproductive organs of red king females consist of ovaries and oviducts. The ovaries are located above the hepatopancreas. The oviducts connect to the ventral sides of the exopodites of the second walking legs, and eggs are extruded via the gonopores [17].

The aim of this study was to determine the fatty acid contents of the testes and ovaries of red king crabs living in the Barents Sea and to compare these profiles with those found in other red king crab tissues.

## 2. Materials and Methods

Red king crabs were taken directly by SCUBA diving along standard transects randomly situated at 5–30 m depth in Dalnezelenetskaya Bay, Southern Barents Sea, Russia (Figure 1), in July 2016. Dalnezelenetskaya Bay is a semi-closed, relatively small gulf, with five islands separating it from the open sea area. The area is considered typical of the eastern coast of the Kola Peninsula [18,19].

In the laboratory, crab sex was determined by direct observation of the abdominal region. The crabs were measured for carapace length, i.e., the line from the posterior margin of the right eye orbit to the medial-posterior margin of the carapace, using digital calipers to the nearest 0.1 mm. Each crab was individually weighed. The shell-age categories of crabs were determined visually according to standard indicators of elapsed time since molting, including the amount of scratching on the ventral surface of the carapaces, legs, and coxopodites; shell color; and spine wear [17].

After biometric parameters were determined, each crab was dissected and a 2–10 g sample of its gonads was taken. The gonads were placed in plastic bags and then frozen. Frozen samples were transported to the laboratory of the Institute of Physiology of Natural Adaptations (Federal Center for Integrated Arctic Research, Arkhangelsk, Russia) for fatty acid assays, as described in our previous papers [11,12,20].

The lipids were saponified and esterified for fatty acid assays, following the method by Folch et al. [21], with modifications. Between 0.3 and 0.5 g of the homogenized sample was placed into a 100-mL glass flask containing 10 mL of a chloroform-methanol mixture (2:1) and a solution of 0.2 mg nonadecanoic acid (C19:0) in chloroform. The resulting solution was then mixed for 30 min and the flask was placed in a thermostat for lipid extraction for 10–12 h at 25 °C. The solution was then filtered into a tube and the mixture of chloroform-methanol was added for a final sample volume of 15 mL. Afterward, 0.74% water solution of CaCl_2_ (3 mL) was added to the tube and placed in a refrigerator for 12 h. Over this period, the solution was stratified into two fractions. The upper fraction with water-soluble impurities was removed with a Pasteur pipette, while the lower fraction was taken for further analysis. Methanol (0.5–1.0 mL) was added to the vial and the content was evaporated to dryness in a vacuum evaporator “Multivapor P12” at 318 mbar and 50°C. Prior to being mixed for 5 min, the extract was dissolved in 0.2 mL of the chloroform-methanol mixture. A mixture containing 1.5% solution of H_2_SO_4_ in methanol (2 mL) was transferred to the tube and the sample was incubated in a water bath at 90 °C for 30 min. The sample was placed in a 10-mL tube. Then, distilled water (0.8 mL) was added and the sample was incubated for 2–4 h at ambient temperature. The upper layer was placed in a 2-mL vial and then evaporated in “Multivapor P12” at 195 mbar and 45°C. The resulting sample (200 μL) was transferred to a gas chromatography vial and the fatty acid methyl esters were assayed using an Agilent 7890A gas chromatograph (Agilent Technologies Inc., Wilmington, DE, USA) equipped with a flame ionization detector. The esters were then separated using a 60 m x 0.25 mm x 0.15 μm capillary column Agilent DB-23. Nitrogen was used as the carrier gas with a flow rate of 1 mL min^−1^. The injection volume for the sample was 1 mm^3^. The injection port was maintained at 270 °C and the flame ionization detector was maintained at 280 °C. The initial column temperature was 130 °C; it was increased to 170 °C (at a rate of 8.5 °C min^−1^), 206 °C (at a rate of 2 °C min^−1^), 220 °C (at a rate of 0.7 °C min^−1^), and finally 230 °C (at a rate of 6 °C min^−1^). A certified mixture of 44 methyl esters of fatty acids Nu Chek Prep Inc 569 B was used to determine the retention time of the sample components and calibration coefficients. The Agilent Chem Station B.04.03 software was used for the identification of fatty acids in the samples by comparison of retention times to those of authentic standards.

Differences in carapace lengths and weights in red king crabs and differences in fatty acid levels between males and females and among size groups were tested using a one-way analysis of variance (ANOVA) or non-parametric Kruskal-Wallis test. Homogeneity of variance and normality assumptions of ANOVA were tested and, unless noted, met before each test. If the assumptions were violated (modified Levene’s test, *p* < 0.05), the data were log_10_-transformed prior to analysis; however, if that did not correct the problem, we used Kruskal-Wallis tests.

Statistical analyzes were carried out using STATISTICA version 6. Data are presented as means ± one SD (standard deviation).

## 3. Results

A total of 48 red king crabs (8 males and 40 females) were collected from Dalnezelenetskaya Bay. All the female crabs and 4 males had new shells (age 2–12 months post-ecdysis), while the remaining 4 males had old shells (age 13–24 months post-ecdysis).

Biometric data for the crabs used in biochemical assays are presented in Table 1.

Male red king crabs had a significantly larger carapace length (df = 1, *F* = 6.91, *p* = 0.009) and were heavier (df = 1, H = 7.65, *p* = 0.006) than female crabs.

A total of 43 fatty acids (including trans-isomers) were routinely found and quantified in the gonads of red king crabs at the study site (Table 2).

The proportions of total saturated fatty acids (SFA), monounsaturated fatty acids (MUFA), and polyunsaturated fatty acids (PUFA) in the ovaries of female red king crabs were 25.9 ± 2.0%, 22.5 ± 2.3%, and 51.6 ± 2.5%, respectively; in the testes, these levels accounted for 35.1 ± 5.7%, 19.1 ± 2.0%, and 45.8 ± 4.5%, respectively.

In almost all cases, absolute concentrations of fatty acids were significantly higher in female gonads than in male gonads (Table 2). In both ovaries and testes, palmitic (C16:0) and stearic (C18:0) acid had the highest concentrations among SFAs, while oleic (C18:1n9c) and palmitoleic (C16:1n7c) acid predominated among MUFAs. The total proportion of n-3 PUFAs was higher in females (42.4%) compared to males (33.6%). An opposite pattern was found for n-6 PUFAs: 9.3% in the ovaries and 12.2% in the testes. The highest concentrations of n-3 fatty acids were registered for eicosapentaenoic (EPA, C20:5n3) (5.47 ± 2.16 mg·g^−1^ in females and 0.72 ± 0.49 mg·g^−1^ in males) and docosahexaenoic (DHA, C22:6n3) (3.46 ± 1.26 mg·g^−1^ in females and 0.57 ± 0.50 mg·g^−1^ in males) acid. Among n-6 fatty acids, the highest value was observed for arachidonic acid (C20:4n6): 1.24 ± 0.46 mg·g^−1^ in females and 0.31 ± 0.18 mg·g^−1^ in males. The n-3/n-6 ratio in the ovaries was 4.7 ± 0.8, while in the testes it was 2.8 ± 0.3.

Total fatty acid concentrations in females at size classes 121–130 (sample size, *n* = 9), 131–140 (*n* = 13), 141–150 (*n* = 14), and > 150 mm CL (*n* = 4) were 23.1 ± 3.0, 25.4 ± 1.7, 19.7 ± 1.8, and 21.2 ± 2.0 mg·g^−1^, respectively. These levels did not differ significantly from each other (df = 3, *F* = 1.56, *p* = 0.216). We also compared the fatty acid levels in the testes of males with new (*n* = 4, 3.5 ± 0.5 mg·g^−1^) and old (*n* = 4, 4.1 ± 1.5 mg·g^−1^) shells and found no significant differences (df = 3, *F* = 0.15, *p* = 0.710).

## 4. Discussion

Our study is the first report on the fatty acid composition in the gonads of red king crab from the Barents Sea. We found that PUFAs had the highest concentrations in the testes and ovaries of *P. camtschaticus* relative to SFAs and MUFAs. A similar pattern has been reported for other marine crustaceans [22,23,24]. In some species, however, the major fatty acids in gonads have been either SFA [25,26] or MFA [27], corresponding to physiological aspects of these crustaceans that occur in different regions, depths, and environments.

The total concentrations of fatty acids in the hepatopancreas, meat, and hemolymph of the Barents Sea red king crabs (combined data for both sexes) have previously been assayed to be 4.1 ± 1.5, 2.9 ± 0.1, and 0.7 ± 0.1 mg·g^−1^, respectively [11,12,15]. These tissue-specific levels were 5, 7.5, and 29 times lower, respectively, than those found in the ovaries (22.0 ± 1.2 mg·g^−1^) in the current study. The testes’ fatty acid level (3.8 ± 0.9 mg·g^−1^) observed herein was similar to the hepatopancreas and meat levels reported by Dvoretsky et al. [12,15], but five times higher than the reported hemolymph level [11]. These variations, undoubtedly, reflect basic differences in functional roles and the physiology of different tissues and organs of red king crabs [17].

Lipids have many roles within the body, acting as energy sources, structural components of membranes, and signaling biomolecules [28]. All of these aspects are important for normal reproductive function [29]. The reproductive cycle of crustaceans includes a series of events such as the proliferation of gonial cells, differentiation and growth of gametes, reproductive processes associated with mating, and incubation of embryos until the release of larvae or juveniles [30]. Crustaceans are known to produce large numbers of yolk-laden eggs and brood them externally for extended periods. In red king crabs, the gonad index (a ratio between the gonad and body weight) of males is much lower than that of females [1]. We found that the total fatty acid concentration in the ovaries was 6 times higher than in the testes. For SFA, MFA, and PUFA, these ratios were 4.5, 7, and 6, respectively. The highest difference (17 times) was detected for palmitoleic acid. Fatty acids are widely associated with the utilization of endogenous lipid resources, particularly for reproductive purposes [30]. For example, some crustaceans use fatty acids in the regulation of ovarian and oocyte maturation [31,32]. Thus, the sex-specific differences observed in the current study in the fatty acid composition of the *P. camtschaticus* ovaries and testes were associated with differences in male and female energy investment in reproduction. According to general trade-off strategies, increased reproductive efforts cause reduced longevity of the parent [33]; thus, the adult female red king crabs used in our fatty acid assays were smaller than the same-aged males due to greater energy allocation to reproduction relative to growth (Table 1).

Higher fatty acid values in the gonads of females compared to males have been reported for other commercial crustaceans, including the Dungeness crab *Cancer magister* from Humboldt Bay, California, USA [34], the edible crab *Cancer pagurus* [23], the orange mud crab *Scylla olivacea* from Setiu Wetlands, Malaysia [24], and the European lobster *Homarus gammarus* from Scotland [22], indicating the lower cost of spermatogenesis compared to the cost of egg production.

We found that the size of the *P. camtschaticus* females did not affect fatty acid concentrations in their gonads. A similar result was found for males with new and old shells. These findings are likely explained by the crabs that were compared having been in the same physiological status: breeding and molting seasons took place 2–4 months before the sampling period [35,36,37].

Dietary n-3 PUFA has been shown to decrease the risk of developing cardiovascular disease in humans and have a number of positive benefits in patients with some disease already [38,39]. The beneficial effects of dietary n-3 PUFA include anti-inflammatory activity, and these substances have been recommended for therapy for rheumatoid arthritis [40] and, to some extent, inflammatory bowel diseases such as ulcerative colitis and Crohn’s disease [41]. Epidemiological research showed that n-3 PUFA may decrease risks in colorectal, breast, and prostate cancers [42,43], and can be beneficial in chemotherapy [44]. Fatty acids have been found to have some rare properties such as antiviral, anti-helminthic, anti-psoriatic, anti-ischemic, and anti-infective activities [45]. As a result, many recommendations for DHA and EPA intake for humans have been produced by government bodies and various national health agencies and associations [46]. Fish and seafood rich in n-3 long-chain PUFAs have been recognized as important components of a healthy human diet worldwide [47]. The high levels of EPA and DHA detected in the ovaries of red king crabs from the Barents Sea in the current study indicate that these gonads can be a good source of these fatty acids in the human diet and/or for extracting important fatty acids for medical and pharmaceutical industries.

## 5. Conclusions

An assay for the fatty acid content of the gonads of male and female red king crabs from the Barents Sea showed that, as in the organs of many other decapod crustaceans, n-3 PUFA was more abundant than SFA and MUFA. We found that size and shell condition did not affect fatty acid concentrations in red king crabs. Ovaries contained higher levels of fatty acids when compared to testes, reflecting a higher investment of females in reproduction. Ovaries have good nutrition quality in terms of high concentrations of essential fatty acids and optimal ratios of n-3/n-6 PUFA, which makes this tissue a potential source of valuable biochemical substances for the pharmaceutical and food industries.

## Figures and Tables

**Table 1:** Size and weight parameters of red king crabs from Dalnezelenetskaya Bay used in fatty acid analyses.

Sex	Carapace Length, mm					Weight, g			
	N	X	SE	Min	Max	X	SE	Min	Max
Female	40	147.6	2.0	101.0	173.5	1810.6	63.2	591	2633
Male	8	174.4	11.9	122.5	230.0	3090.1	631.6	980	6320
Female + Male	48	152.0	2.9	101.0	230.0	2023.9	132.3	591	6320

Note: N—sample size, X—mean, SE—standard error, Min—minimum, Max—maximum.

**Table 2:** Fatty acid composition in the gonads of red king crabs from Dalnezelenetskaya Bay, Barents Sea, Russia.

Fatty Acid	Concentration, μg·g^−1^				% of Total Fatty Acid				*p*
	Female		Male		Female		Male		
	X	SD	X	SD	X	SD	X	SD	
C6:0	1.9	1.2	1.0	0.2	0.009	0.008	0.032	0.012	0.115
C8:0	2.1	0.7	2.5	1.0	0.013	0.017	0.084	0.041	0.185
C9:0	2.2	0.8	2.5	1.1	0.012	0.011	0.081	0.041	0.379
C10:0	2.9	1.6	2.4	1.1	0.017	0.016	0.079	0.040	0.342
C11:0	1.8	1.0	2.1	1.3	0.010	0.008	0.067	0.042	0.460
C12:0	49.2	14.6	15.0	6.8	0.248	0.100	0.463	0.253	<0.001
C13:0	7.3	2.1	2.3	0.7	0.036	0.012	0.073	0.032	<0.001
C14:0	348.8	154.9	45.7	16.0	1.536	0.295	1.316	0.233	<0.001
C15:0	169.5	68.0	23.3	10.5	0.767	0.181	0.652	0.102	<0.001
C16:0	3463.8	1254.4	672.3	339.5	15.776	1.249	18.445	2.626	<0.001
C17:0	132.2	40.8	33.8	20.9	0.628	0.128	0.898	0.113	<0.001
C18:0	1172.7	355.7	406.1	160.9	5.648	1.392	11.621	2.366	<0.001
C20:0	53.9	23.6	15.0	4.7	0.245	0.092	0.438	0.082	<0.001
C21:0	12.7	10.0	2.9	0.8	0.056	0.034	0.092	0.031	<0.001
C22:0	16.5	8.1	6.0	2.0	0.079	0.040	0.187	0.071	<0.001
C23:0	83.2	59.9	9.3	7.6	0.356	0.220	0.228	0.118	<0.001
C24:0	94.5	57.8	15.0	11.0	0.424	0.208	0.383	0.082	<0.001
C14:1n5t	–	–	2.9	0.0	–	–	0.005	0.012	–
C14:1n5c	4.3	1.8	2.4	1.8	0.020	0.008	0.071	0.059	<0.001
C15:1	13.6	18.1	5.1	5.3	0.039	0.067	0.068	0.121	0.411
C16:1n7t	34.4	18.2	8.9	8.1	0.159	0.079	0.209	0.092	<0.001
C16:1n7c	1538.8	752.9	91.7	44.1	6.614	1.793	2.533	0.727	<0.001
C17:1	3.0	2.2	1.6	1.3	0.014	0.012	0.051	0.055	<0.001
C18:1n9t	90.0	32.9	18.9	5.7	0.431	0.139	0.564	0.151	<0.001
C18:1n9c	2709.1	979.7	490.1	313.7	12.453	1.577	12.922	1.280	<0.001
C20:1	552.2	260.1	93.6	83.7	2.436	0.611	2.291	0.851	<0.001
C22:1	50.0	26.2	8.5	5.7	0.223	0.073	0.234	0.086	<0.001
C24:1	29.1	15.1	4.2	1.8	0.130	0.045	0.122	0.028	<0.001
C18:2n6t	36.0	33.7	15.2	9.4	0.164	0.154	0.400	0.123	<0.001
C18:2n6C	275.9	105.0	58.7	46.8	1.283	0.277	1.488	0.252	<0.001
C18:3n3	91.6	44.2	10.7	14.4	0.409	0.125	0.225	0.130	<0.001
C18:3n6	53.2	21.8	7.7	7.8	0.245	0.071	0.180	0.054	<0.001
C20:2n6	208.0	73.5	46.8	32.1	0.976	0.171	1.200	0.177	<0.001
C20:3n6	35.1	17.7	5.2	3.2	0.156	0.037	0.137	0.036	<0.001
C20:4n6	1240.4	464.5	311.2	184.6	5.824	1.218	8.179	1.195	<0.001
C22:2n6	2.4	1.4	0.7	0.2	0.011	0.005	0.021	0.007	<0.001
C20:5n3	5465.4	2160.6	721.3	486.9	24.448	3.067	18.429	2.005	<0.001
C22:6n3	3463.1	1255.2	566.0	497.8	15.911	2.375	13.833	2.818	<0.001
C20:3n3	46.5	25.9	7.2	5.8	0.211	0.066	0.181	0.076	<0.001
C22:4n6	69.7	30.3	12.6	5.3	0.315	0.074	0.362	0.132	<0.001
C22:3n3	2.2	1.9	0.4	0.0	0.010	0.008	0.003	0.008	<0.001
C22:5n6	63.6	23.7	8.5	6.7	0.292	0.084	0.212	0.080	<0.001
C22:5n3	305.8	130.8	34.7	19.3	1.364	0.220	0.939	0.334	<0.001
∑SFA	5611.7	1903.6	1257.3	568.3	25.861	2.006	35.139	5.667	<0.001
∑MUFA	5018.8	1940.5	723.4	458.2	22.520	2.273	19.070	1.955	<0.001
∑PUFA	11357.9	4004.0	1806.7	1297.3	51.620	2.463	45.791	4.548	<0.001
Total	21988.4	7732.4	3787.3	2307.9	100.000	0.000	100.000	0.000	<0.001
∑n-3	9374.5	3363.5	1340.0	1013.2	42.353	2.290	33.611	4.063	<0.001
∑n-6	1983.4	703.6	466.6	287.4	9.266	1.433	12.180	1.054	<0.001
∑n-9	3430.4	1273.6	615.4	408.0	15.674	1.903	16.133	2.142	<0.001
∑n-7	1575.8	760.3	102.3	51.1	6.787	1.794	2.793	0.663	<0.001
n-3/n-6	4.7	0.8	2.8	0.3	4.682	0.777	2.767	0.333	<0.001

Note: X—mean, SD—standard deviation, SFA—saturated fatty acids, MUFA—monounsaturated fatty acids, PUFA—polyunsaturated fatty acids, *p*—p-value for ANOVA comparisons between absolute fatty acid concentrations in male and female crabs.

## Data Availability

The data are available upon request from the corresponding author.
